# How can I help at this moment? Outlining three generations of coaching for health professions educators

**DOI:** 10.1111/medu.70142

**Published:** 2025-12-23

**Authors:** Rune D. Jensen, Ingrid Price, Kevin W. Eva

**Affiliations:** ^1^ MidtSim, Department of Clinical Medicine Aarhus University Aarhus Denmark; ^2^ Faculty of Pharmaceutical Sciences University of British Columbia Vancouver British Columbia Canada; ^3^ Department of Medicine and Centre for Health Education Scholarship University of British Columbia Vancouver British Columbia Canada

## Abstract

**Background:**

Coaching is increasingly recognised as a valuable tool in health professions education (HPE), supporting learning, performance, and well‐being. Yet, the term ‘coaching’ is used inconsistently, leading to confusion and limiting its potential impact. In this cross‐cutting edge article, the authors draw upon a framework from the sport psychology and organisational development literatures to outline three distinct generations of coaching that can guide HPE away from treating coaching as a unitary construct. In doing so, we strive to clarify coaching's varied purposes and paradigms.

**The Three Generations Framework:**

Generation 1 emphasises performance management, focusing on goal‐setting and problem‐solving. Generation 2 shifts towards personal development, leveraging strengths and fostering long‐term growth. Generation 3 centers on meaning‐making and cultural transformation, prioritising values, identity and reflective dialogue. The authors argue that each generation offers unique benefits and limitations and that coaching in HPE should be deliberately aligned with the learner's context and goals. Through examples from clinical education, they illustrate how different coaching approaches can support technical skill development, professional identity formation and well‐being.

**Implications:**

Rather than advocating for a single model, the article encourages educators to adopt a flexible, context‐sensitive approach to coaching. By understanding the generational distinctions, HPE professionals can better tailor coaching interventions to meet learners' evolving needs and foster sustainable development. As coaching continues to expand in scope and complexity, this framework offers a timely lens for enhancing clarity, intentionality, and impact in health professions education.

## Abstract 2

Need help determining how to coach colleagues or trainees? In this manuscript three generations of coaching strategy are detailed with guidance as to when to apply one or another. #MedEd

## 1 INTRODUCTION

Recent years have seen a proliferation of coaching studies in health professions education, for good reason: Various publications have shown that coaching can facilitate learning, well‐being, and performance among health care professionals.^1^, ^2^, ^3^ However, like every such proliferation before it,^4^ concern arises about variability in the term's use, the resultant risk of miscommunication and suboptimal intervention if clarity is not achieved. In actual fact, there are many models of coaching that have been proffered, each with distinct purpose, logic, theoretical framing and emphasis. It is not necessarily the case that one is better than the other; rather, different models may be appropriate in different contexts or at different moments in time. Unfortunately, the distinctions between models and their implications have not been detailed explicitly in health professions education. In this cross‐cutting edge article, we draw upon the sport psychology and business literatures to outline three generations of coaching, offering health professions educators and trainees a more concrete means of specifying, which is most likely to fit their workplace‐based learning goals; what they might hope to achieve; and what limitations underlie the paradigms supporting each generation. To do so, we detail the work of Grant^5^ and Stelter^6^ because they have been particularly prominent in documenting the evolution of coaching. We then draw connections between the roots of coaching research and how it has been applied in health professional education research and practice contexts. Before getting into those details, however, some definitional foundations must be laid.

## 2 FOUNDATIONS OF COACHING

‘Workplace coaching’ (referred to simply as coaching hereafter) describes the application of effort, within workplace contexts, often by a supervisor or manager, to guide learners or employees towards the attainment of work‐related goals. Given the breadth of activity the term encompasses, it is easy to understand why coaching is often confused with related constructs. It is not ‘teaching’ in the sense of imparting knowledge, but its activities span from formal structured sessions that might involve teaching to informal ad‐hoc conversations that are integrated into daily work interactions.^5^ Nor is it ‘feedback’ in the sense of implying judgement about what aspects of performance should be improved, but its activities span from voluntary coachee‐led conversations to interactions that arise due to remedial activity being required.^5^ The boundaries in relation to ‘mentoring’ are particularly unclear.^2^ Often seen distinctions include the following: (a) Field‐specific knowledge, experience, and wisdom are generally expected of mentors, but not necessarily of coaches^7^; (b) the ‘end‐game’ of mentoring is generally to become like the mentor while coaching is primarily pursued to help the coachee become a better version of themselves^8^; and (c) coaching often holds a longer term focus on career development than mentoring, which is often undertaken to address a particular need.^9^ Here, we will not fall into the trap of trying to offer a single universal definition of coaching but strive to highlight differences in the way the term is used that arise dependent on how, when and where one acts for the sake of helping another pursue learning goals.

Reports of coaching began to arise in the management literature in the 1980s. Here, coaching generally referred to optimising human performance,^5^ but how the phenomenon of coaching was approached (and, hence, what methodologies were promoted) branched soon afterwards. One view, anchored in sport psychology, placed strong emphasis on performance; the other, anchored in humanistic and existential psychology, focused more heavily on self‐actualisation (i.e., realising one's full potential).^10^, ^11^ Since the use of coaching began to broaden in the 1990s, Grant^5^ and Stelter^6^ have been scholarly leaders in drawing attention to distinct worldviews regarding coaching, its purpose and its value. In doing so, they document three generations of thought that remain in contemporary use. As such, we use their three generations for guidance about how health professions education can use the term coaching with more nuance and intentionality by understanding how practice has evolved in other domains (see Table 1). It is important to acknowledge that there is overlap between the generations, making them less clearly distinct temporally than the word ‘generation’ might imply. While it is true that people working in different domains may have embraced or abandoned specific approaches, understanding coaching from the generational framework offers a valuable perspective on evolving trends. Thus, to facilitate decision‐making regarding what form of coaching might best help in a given context, we offer a brief review of each generation before turning attention to how that evolution aligns with the way coaching is discussed and practiced in health professions education.

**TABLE 1:** Characteristics of coaching in each of three generations identified by Grant^5^ and Stelter^6^ (presented with the caveat that considerable overlap will exist in practice).

Dimension	First generation	Second generation	Third generation
Focus	Performance management Fix weak performance Skill development	Personal development Leveraging strengths of all Thoughts and feelings	Meaning‐making/well‐being Reflective dialogue Values and priorities
Overarching aim	Solving a particular problem to meet a standard that is external to the coachee	Enabling the coachee to leverage their assets to make change driven by their desires	Enabling the coachee to align their values/core beliefs to make change that aligns with their social context
Example	GROW model^12^	Appreciative inquiry^14^	Narrative‐collaborative practice^21^
Locus of control	Coach	Coachee strengths	Coachee values and priorities
Motivator	Professional expectation	Individual career goals	Situation suggestive of broad issue
Typical data source	Observation of coachee	Conversation between coach and coachee	Indications of under‐performance or dissatisfaction
Key activity	Developing learning goals	Structuring action plans	Collaborative dialogue
Required asset	Capacity to perform the desired skill/offer insight	Capacity to facilitate reflection	Capacity to build trust and psychological safety
Particular use	Skill development around specific/tangible problem (often with right/wrong answers)	More complex problems related to career direction (often less concrete)	Engendering receptivity from the coachee (often pertaining to power imbalance)
Clinical education example	Improving debriefing skills following clinical procedure	Improving supervision to become education leader	Overcoming poor teaching evaluations
Critique	Metric chasing Culture of fear/anxiety Overlooking importance of relationships and values	Lack of relevance to the workplace Jargonised language Demotivating if progress not recognised	Disconnect from specific needs Risk of building psychological barriers Risk of ‘othering’ coachee

## 3 GENERATION 1: COACHING AS PERFORMANCE MANAGEMENT

Starting in the late 1980s and throughout the 1990s, first generation models of coaching were heavily focused on performance management.^5^ That is, the dominant aim of coaching was to identify a problem and enable managers to coach others to overcome it.^15^ The GROW model, published by Sir John Whitmore in *Coaching for Performance*, arose during this generation.^16^ The method begins with coaches helping their coachee identify a problem (the ‘**G**oal’ they want to pursue). It is the need to do so that led Whitmore to propose the still popular SMART criteria in coaching, which prioritise goals that are Specific, Measurable, Accepted, Realistic and Time‐based.^12^ Once a goal is identified, the coach's role is to encourage the coachee to reflect on their ‘**R**eality’ (assessing their current ability and context), to brainstorm the ‘**O**ptions’ available to improve and to choose from this list of options what they ‘**W**ill’ commit to doing.

Key to understanding first generation coaching is locus of control. As Grant states, ‘the workplace coaching literature at this point in time was almost invariably associated with managing poor performances’.^5^ Coaches, in other words, were individuals who were responsible for identifying ‘difficult’ employees, often through mandated performance reviews, and for figuring out how to manage them towards overcoming problems. As the GROW model exemplifies, the coachee was expected to take an active role, but coaching was generally initiated by and focused on problems identified by the coach. As one empirical example, consider a study in which sales managers were engaged in a 5‐day course aimed at training them to assume a coaching role.^17^ The managers were expected to coach employees with the lowest numbers of sales towards better performance. While observing the salesperson's interaction with a client, managers were taught to use a behavioural checklist to identify improvement needs. Upon observing a net increase in sales, the authors concluded that coaching created clarity regarding performance expectations, enabled feedback to be given and provided rewards that were motivating.

While such benefits may be achieved, first generation coaching and its emphasis on goal‐setting and problem‐solving have been critiqued for its potential to inadvertently encourage employees to pursue unethical methods to attain performance targets such as misrepresenting their performance levels.^18^, ^19^ The culture of fear and anxiety accompanying Generation 1 coaching often led to burnout that eventually led organisations away from managing through a crisis mentality.^5^ That said, goal‐focused approaches like the GROW model are still useful and extensively applied^20^; now, however, they are more often used with aims to leverage each coachee's strengths and identify their own goals rather than simply pushing coachees towards a desired solution, consistent with the development of Generation 2.

## 4 GENERATION 2: COACHING AS TALENT MANAGEMENT

With the dawn of the 21st century and the advent of the knowledge economy, organisations began to see a rise in competition to attract and retain skilled workers. Such pressures, along with the limitations of Generation 1, led coaching advocates to shift towards efforts to enhance the strength of all employees rather than focusing on weaknesses that needed to be addressed for a few. In other words, growth and development for the long‐term began to take priority with increased attention paid to consultation with the coachee about their needs.^21^, ^22^ Positive psychology coaching and appreciative inquiry coaching (AIC) provide good examples of the strength‐orientation underlying Generation 2. In the former, emphasis is placed on building positive emotions, engagement, and meaning‐making for the coachee to motivate them to pursue stronger performance.^23^ AIC similarly shifts the conversation about change by exploring areas of success and strength as a lens through which to consider solutions to a particular problem.^14^

While the ultimate goal of second generation coaching remains performance improvement and still prioritises teaching managers to ‘sit down and converse’ to solve specific problems, emphasis shifted to greater degrees of humanism as constructs such as Emotional Intelligence were layered on.^5^ That is, first generation coaching focuses on what the coachee lacks, their areas of weakness and creating a strategy to resolve it; second generation coaching seeks to leverage the coachee's particular strengths to not just overcome the problem but to enable strategy for personal development. In a meta‐analysis on the use of AIC, Bushe and Kassam found that, for AIC to be transformative, it should be used to (a) change how people think rather than focus on what they do and (b) focus on supporting change processes that flow from new ideas.^24^ When applying AIC, it is important to not get blinded by the ‘positive stuff’, as Bushe framed it.^25^ Rather than promoting enchantment, the main purpose of AIC is to enable spontaneous action towards a better future. Focusing on the positive, in other words, is the means for reaching the goal of generating new and better practice, inducing open‐mindedness over limiting focus on the current problem.

Despite such promising results, several problems arose from second generation coaching when it was initially practiced. The broadened emphasis, often valuable when coaching executives, was found to have less relevance for many workplace‐based tasks; highly jargonised language divided people into in‐groups and outgroups characterised by ‘evangelical zeal’ and disenchanted resentment, respectively; and emphasis on formally structured conversations tended to feel disconnected from workplace constraints.^5^ Most core, however, is that emphasis in both Generation 1 and Generation 2 is on individual achievement of goals within the current system whereas changing organisations required consideration of how coaching might influence culture, leading to Generation 3.

## 5 GENERATION 3: COACHING AS CULTURE CHANGE

Third generation coaching prioritises well‐being and meaning‐making in addition to performance of the individual. While individual goals can and are still achieved, the focus of coaching is much deeper than Generation 1 or 2, encouraging the coachee to explore what a meaningful life looks like for them as a means to define goals and make decisions associated with these. As such, Stelter argues that rather than looking at problems or successes, third generation coaching emphasises flexible dialogue with the goal of strengthening clients' ability to reflect: ‘… making meaning occurs when we attribute certain values to our experiences, actions, interactions with others and our personal and professional circumstances’.^13^ In doing so, coachees develop the ability to focus on the ‘bigger picture’ and engage in self‐regulation that enables sustainable coping in the face of varied challenges. Good coaching, in other words, is less about the adoption of any particular model than it is about agency, intentionality, agility, and flexibility. Relatedness between the coach and the coachee is, thus, critical as coachees need to be attracted by opportunities to contemplate changes that align with their values.

A key element in Generation 3 is for the coach to focus dialogue around an experience, raising questions about why it is important rather than what was done.^26^ To establish such, Stelter and Law^21^ developed a methodology that encourages coaches to engage in ‘narrative‐collaborative practice’, treating both coach and coachees as experts in their own rights. To this dialogue, coachees bring aspirations and a sense of purpose towards action that has meaning to them and reflects their own values; the coach's role, in contrast, is to help refine self‐understanding and goal pursuits. Godskesen and Kobayashi used those principles to illustrate that coaching could improve supervisor‐student relationships and enhance students' feelings of progress.^27^ Similarly, a randomised controlled trial found that value‐based coaching could improve trainee well‐being.^28^ As evidence accumulates that coaching for awareness of one's emotions and thoughts can enable students to become more reflective, mindful, and self‐determined,^29^ we see how outcomes of value‐based coaching are related to a broader array of concepts than capacity to overcome a specific problem.

That is not to say that Generation 3 coaching ‘solves’ the problems of Generations 1 and 2; nor is it a replacement for these approaches. In fact, it is easy to imagine coachees being frustrated by holistic coaching efforts when they are particularly focused on trying to solve a particular problem. Similarly, the array of constructs needed to describe Generation 3 likely replicates the problems of disenchanting some through jargonised language. Considering the generations of coaching as complementary raises ‘bigger picture’ questions about when each is valuable. That is, what features of varied circumstances might help a particular coach prioritise a specific strategy? How can I help *at this moment?* Similarly, what features might help one conclude that a different coach would be a better fit for the demands of the situation? Am I the right person to help *at this moment*? To begin to grapple with such questions we offer a brief summary and examples of how coaching has been described in health professional education.

## 6 HEALTH PROFESSIONAL EDUCATION AND THE COACHING GENERATIONS

In health professions education, coaching has taken on increasing degrees of prominence. Many have been led to its value in hopes of enabling self‐regulating professionals to engage in lifelong quality improvement efforts and to separate trainees' efforts at professional development from the threats accompanying feedback and assessment.^30^, ^31^, ^32^ Little concerted effort, however, has been exerted towards comparing and contrasting the various forms of coaching, preventing careful analysis of whether efforts are effectively tailored to their purpose.

Lovell's review highlighted coaching's great promise for personal and professional development in medical education while noting complications arising from theoretical uncertainty that reinforce the need for a detailed taxonomy of coaching models such as that outlined in this article.^2^ The author concluded that the evidence for coaching's benefits was strongest for teaching technical skills, relative to non‐technical skills or coaching for well‐being/resilience. For example, Bonrath and colleagues utilised behavioural modelling to address deficiencies and enhance positive behaviours in laparoscopic surgery by presenting both good and poor examples to coach an intervention group, which achieved better surgical performance than control participants.^33^ Such findings, however, need to be contextualised by recognising that the focus of coaching in Lovell's review involved behavioural observation and feedback, with the coach having expertise in the relevant context.^2^ That definition, which seems very much in alignment with a Generation 1 perspective, limited the author's inclusion criteria to behaviourally focused coaching interactions. Indeed, none of the 21 papers reviewed focused on the relationship‐centred or value‐based interactions that would align with second or third generation coaching. Therefore, this article provides a good example both of how Generation 1 can be useful for skill development and why the evidence uncovered by Lovell may have been weaker for coaching supportive of non‐technical skills or well‐being.^2^

An example of Generation 2 coaching, focusing on strengths to achieve desired goals, can be found in work by Palamara and colleagues, published in the same year as Bonrath et al. In this study, coaching aimed to minimise burn‐out and promote success during internal medicine residency.^34^ Residents were partnered with faculty members trained as coaches using positive psychology exercises. The programme offered residents four coaching sessions that employed structured activities directed towards accessing positive emotions and strengths as a means to achieve goals residents set for themselves. In doing so, the authors report more wellness improvement than would be expected from the conclusions of Generation 1 style coaching intervention studies.

More recently, and particularly useful for furthering conversation regarding aligning one's coaching to fit the circumstances, is the popular R2C2 model.^35^ Based on facilitated reflective performance feedback, R2C2 describes 4 phases: build **R**elationship, explore **R**eactions to performance data, explore understanding of the data **C**ontent and **C**oach for performance change. The model primarily emphasises developing learning goals. Therefore, dependent on the focus of the coaching, R2C2 could be considered Generation 1. The conversations promoted, however, can focus on the coachee structuring action plans to find their own solutions based on their strengths, which aligns more with Generation 2. Further, an explicit component of the R2C2 model involves collaborative dialogue and reflective practice, aiming to develop the coachee's self‐awareness and meaning‐making abilities, which resides in Generation 3. As alluded to early in this article, while feedback can be part of the coaching dialogues, it is not a fundamental expectation. Depending on the context, flexible incorporation of feedback or withholding of such may be important to maintain coachee receptivity and engagement.^36^

In exploring R2C2 in practice, Armson et al. differentiate between process‐oriented and content‐oriented coaching skills.^37^ In doing so, they observed that the way in which coaches prepare, develop relationships with coachees, and communicate (process‐oriented coaching skills) is interconnected with content‐oriented coaching skills such as offering feedback, collaborative goal setting, and co‐development of learning plans. They report, thus, that fundamental tensions exist between encouraging self‐direction and ensuring competence; both are deemed important but cannot necessarily be achieved at the same time. Finding the right balance, therefore, between ‘coaching dialogue and teaching monologue’ appears to depend heavily on the particular goals to be achieved, demanding more careful consideration of the advantages and disadvantages of any coaching generation.

## 7 IMPLICATIONS

### 7.1 How to choose?

According to both Grant and Stelter, it is important to be deliberate and aware of when coaching should focus on resolving problems versus personal development versus meaning‐making and well‐being.^5^, ^6^ Aligning needs and expectations with the coaching approach, after all, is critical for maintaining coachees' motivation, engagement and perceived value. Their work suggests that Generation 1 is particularly useful for skill development and giving feedback around a specific and tangible problem that resides in the individual. Such activities are often recognisable because they contain right/wrong answers and, thereby, can be reasonably expert‐led. Generation 2 is more relevant for more complex problems such as what direction an individual wants to take their career by identifying and leveraging their strengths. Finally, Generation 3 may be particularly valuable when there is a need to engender receptivity on the part of the coachee such as when there is a power imbalance, a need to carefully manage a relationship or identity threat.

To unpack these distinctions, summarised in Table 1, and offer reflection on the operationalisation, advantages and disadvantages of each generation, consider a surgeon who wants to improve their ability to supervise trainees.

If the surgeon wishes to improve their debriefing skills with trainees after watching them perform a surgical procedure, they may benefit from coaching using a first generation model. This could include the coach observing a debrief session in which they focus on the surgeon's behaviour, recording markers that may relate to problems encountered. The coach then shares their observations with the surgeon during a later coaching session, providing the opportunity to explore the alignment between what the surgeon intended and what the coach observed as a means to identify options to enhance the surgeon's debriefing skills. That is, a coach working within this generation would focus predominantly on a specific problem that the coachee wishes to resolve. The relationship between the coach and coachee can be largely immaterial in such settings because the goal is easily definable; what is up for debate may simply be the best way to get there. Benefits of adopting Generation 1 include the facility to induce specific and clear impact. Drawbacks, however, include the potential to overlook the importance of relational and value‐based dynamics that accompany Generations 2 and 3. That may influence the extent to which the surgeon is motivated to prioritise improving when other issues distract from their desire to perform well, when they disagree with their coach's assessment or when the problem being addressed is a superficial symptom of an unrecognised deeper issue.

On the other hand, if the surgeon wants to enhance their supervision of trainees out of desire to become an educational leader, coaching using a second generation model may be of benefit by drawing the coachee's attention to what has worked well for them and their colleagues in similar situations and/or when they felt energised by their supervision experiences.^3^ That is, a coach working within this generation would help pinpoint positive markers that reinforce the desired behaviours. Doing so engages the surgeon to identify and amplify their unique strengths as an instructor in an effort to make such behaviours more commonplace. Here, the relationship between coach and coachee becomes more important because the coach's role is, in part, to inspire the surgeon educator to work towards change by exploring successes and challenges they have faced to illuminate their unique path towards their goal. Further, the focus of change is based on the strengths of the coachee rather than solving a more concrete problem, like improving their capacity to evaluate a trainee. In this form of coaching, the coach does not need to have the skill to perform or judge the quality of the surgeon's success in supervising learners, but should focus on facilitating a beneficial degree of reflection on their part. It can be beneficial to keep the focus on how different forms of strength might yield the coachee's aim rather than considering there to be ‘one right way’ to enhance performance. Drawbacks, however, include the risk of losing the coachee's interest and patience if progress towards bigger picture goals is not readily recognisable to the degree expected with Generation 1 coaching.

Finally, a surgeon focused on improving supervisory skills because they have received poor teacher ratings might reasonably need a coach to provide support using a third generation model. Generation 3 prioritises a more holistic, integrative approach to coaching that invokes the coachee's values and how they align with improving supervision skills. By taking this approach, the conversation moves towards what is important to the coachee, mitigating the threat created by the poor ratings and making it easier to overcome personal barriers such as professional identity conflicts or a perceived risk of practice constraints. To achieve a level of conversation that holds greater meaning and value to the coachee, the coach must make extended efforts to build a strong relational foundation and a safe, trusting, and open environment where the surgeon feels comfortable discussing their challenges. Such coaching sessions, therefore, generally involve a narrative dialogue to explore the surgeon coachee's experiences and feelings, emphasising strategies they might undertake to change their behaviour in a way that aligns with their priorities and values. Benefits of this form of coaching include helping coachees achieve deeper appreciation for the limitations of their previous approaches, enabling generalisation to other aspects of performance, and building a greater sense of relatedness with their coach and colleagues, which can contribute to a stronger sense of belonging and well‐being. Drawbacks, however, include the risk of building psychological barriers or a sense of being ‘othered’ if a sense of trust is not established, resulting in the coachee being unable to overcome defensive reactions or superficial solutions.

In sum, it is fitting that Armson et al. conclude that ‘effective coaching by supervisors requires a combination of specific process and content skills that are chosen depending on the needs of the individual’.^37^ An effective coach must be able to recognise when each generation of coaching is best utilised and either adapt their approach or refer the coachee to someone else who is a better fit. Such adaptability increases the likelihood that coaching is comprehensive, addressing both immediate performance issues and fostering long‐term development and personal growth as needed and opportunity allows. By balancing these aspects, coaches can provide more nuanced and effective support, ultimately leading to better outcomes for the people they are guiding.

### 7.2 How to implement?

To ensure a coaching relationship is successful, it is important to articulate not only the goal of the coaching relationship but also how best to achieve it. Common to all coaching generations is a focus on positive change (i.e., growth). A central tenet to coaching (regardless of the model employed), therefore, is that the goal is based on what a coachee needs relative to ‘where they are’. Everything a coach does that qualifies as coaching is in service to that outcome. Still, differences in implementation arise dependent on what generation one is coaching within.

If the change involves developing a particular skill and Generation 1 is, thus, the best fit, a coach is needed because they can offer insights the coachee does not yet have (by virtue of lack of expertise or difficulties inherent in overcoming blindspots). To support proficiency development, the coach must have insight into the behaviour the coachee wishes to change. The process through which such insight is gained may, therefore, involve observation of the coachee practicing the skill and engaging in a feedback cycle. It is likely of minimal consequence to the success of coaching whether or not the coach and coachee form a strong relationship given that the focus is on something that is expected to reside in the coachee's professional repertoire.

If the change the coachee desires involves a shift in thinking, managing emotional responses to change behaviour or pursuing longer term career goals and Generation 2 is, thus, the best fit, the central question for coachees becomes ‘how can I leverage my strengths to make this change?’. The coachee is still motivated to improve in some way, but the coaching focus is not on practicing a skill per se; it is about identifying strengths and removing obstacles impinging upon their ability to grow and progress. To support professional development, the coach may need to help the coachee recognise the difference between superficially apparent challenges and deeper conceptualisations that may more fundamentally be holding them back. Using the example above, it may not be capacity as an educator that is holding the surgeon back from becoming an education leader, but a misunderstanding of what it takes to make that transition. Compared to Generation 1, it is less essential that the coach be skilled in the area in which the coachee works. The coach, however, still needs to help the coachee refine their alignment with the culture or profession they generally function within. That is, one must have the skills required to help coachees explore their reality and facilitate identification and resolution of their own issues, connecting authentically in a way that reinforces ongoing efforts. Generation 2 coaching, thus, likely requires longer and deeper conversations regarding the reality of the situation and viability of options for moving the coachee forward because issues are less overt and, hence, solutions less obvious. For foci more aligned with Generation 2, it can be difficult for coachees to be aware of what is holding them back given human tendencies to get locked into a particular way of thinking, generating blind spots that impede capacity to see alternative solutions.

If the change desired by, or required of, the trainee reflects meaning‐making, a values‐based process, then Generation 3 coaching may be more useful because it offers opportunity to shift away from how the coachee can fit into a pre‐established role towards encouraging reflection on what is important to the coachee with respect to their work and life choices more generally. The central questions related to this generation are ‘What are my values & priorities?’, ‘How can I live in accordance with these?’, ‘How can I create meaning in my work?’ and ‘What does a well‐lived life look like for me?’. The outcomes of coaching to be pursued within this generation involve establishing prioritisation and, thus, often require recognition that a specific problem is merely an indicator of a broader issue. It may be beneficial for the coach to be a distant observer in these cases because indications of under‐performance are often the initiating event that prompts such reflection and trust/safety is vital. Continuing the above example, the surgeon may be struggling with teaching because it is simply not their priority. This is why Stelter and Law offer a narrative approach to coaching while arguing that the more neutral the coach is the more effective they can be in supporting coachees to become aware of and reauthor their narrative.^21^

In all forms of coaching, trust is essential, as a coachee must believe their coach has their best interests at heart rather than competing interests or bias.^38^ For Generation 1 and 2 coaching goals, the coach and coachee are implicitly aligned because the change desired is established by the profession or desired by the coachee. Generation 3 coaching goals, in contrast, often derive from situations that create considerable risk of misalignment if the coach is part of the system within which the coachee is struggling in some way. Even though the coach may be completely supportive of the coachee, their being ‘in the system’ can make it impossible for the coachee to shift away from feeling threatened to explore deeper issues that align with their values to identify a way forward. Godskesen and Kobayashi argue that external coaches enable room for reflection and allow the coachee to share otherwise sensitive matters, revealing doubts and showing weaknesses without the potential for repercussion.^27^

As should be evident through this discussion, there are as many similarities as differences between the three generations. However, variation in the dialogue and the processes to be undertaken requires a significant level of skill and flexibility on the part of the coach. The need for such can be easily overlooked if deliberate attention is not paid to when, why, and how coaching is engaged rather than treating ‘coaching’ as a unitary construct. Further, one should not lock into a particular approach forevermore given that the reason the coachee initially seeks coaching may be different than what their ultimate goal becomes. Most often, individuals choose to be coached because they want something to change; becoming aware of what that change is or what they need to do differently to enable it is part of the coaching process. As such, coaching itself must remain agile, adapting as the reality of the situation comes into finer focus. Similarly, it is imperative that research projects in HPE that include a coaching intervention are deliberate as to what type of coaching is being used to align the investigation with desired outcomes and continue to build a more nuanced understanding through empirical efforts.

As further guidance towards a research agenda for health professional education, we would also note limitations inherent in this article. The framework was primarily derived from the business and psychology literatures, making the application to health professional education interpretive. While we offer illustrative examples, the means through which the taxonomy can be put into practice demands further systematic study. Further, there are many additional theoretical foundations such as self‐regulated learning theory,^39^ adaptive expertise^40^ and professional identity formation^41^ that warrant consideration but are beyond the scope of this article.

## 8 CONCLUSION

In this cross‐cutting edge paper, we outline how thinking about coaching has evolved. In doing so, we argue that all three generations of coaching have a place in health professional education. However, an awareness of the different purposes, logics, and paradigms might be useful when scholars and educators in health profession education strive to utilise or investigate coaching. It is particularly important to embed such considerations into our thinking now as planetary, technological, and society changes begin to increase the complexity and expectations of practice and, thus, makes the focus and likely impact of coaching more variable.^42^

## AUTHOR CONTRIBUTIONS

RD, IP, and KE all contributed to the planning, conceptualization, writing and revision of this manuscript. Each accepts responsibility for its content.

## CONFLICT OF INTEREST STATEMENT

None of the authors have a conflict of interest to disclose.

## ETHICS STATEMENT

Not applicable.

## Data Availability

Data sharing is not applicable to this article as no datasets were generated or analysed during the current study.
